# Factors associated with mortality in patients with drug-susceptible pulmonary tuberculosis

**DOI:** 10.1186/1471-2334-11-1

**Published:** 2011-01-03

**Authors:** Payam Nahid, Leah G Jarlsberg, Irina Rudoy, Bouke C de Jong, Alon Unger, L Masae Kawamura, Dennis H Osmond, Philip C Hopewell, Charles L Daley

**Affiliations:** 1 Curry International Tuberculosis Center, University of California, San Francisco, CA, USA; 2 The Tuberculosis Control Section, Department of Public Health, San Francisco, CA, USA; 3 New York University, New York, NY, USA; 4 University of California, Los Angeles, USA; 5 Division of Mycobacterial and Respiratory Infections, National Jewish Health, Denver, CO, USA

## Abstract

**Background:**

Tuberculosis is a leading cause of death worldwide, yet the determinants of death are not well understood. We sought to determine risk factors for mortality during treatment of drug-susceptible pulmonary tuberculosis under program settings.

**Methods:**

Retrospective chart review of patients with drug-susceptible tuberculosis reported to the San Francisco Tuberculosis Control Program from 1990-2001.

**Results:**

Of 565 patients meeting eligibility criteria, 37 (6.6%) died during the study period. Of 37 deaths, 12 (32.4%) had tuberculosis listed as a contributing factor. In multivariate analysis controlling for follow-up time, four characteristics were independently associated with mortality: HIV co-infection (HR = 2.57, p = 0.02), older age at tuberculosis diagnosis (HR = 1.52 per 10 years, p = 0.001); initial sputum smear positive for acid fast bacilli (HR = 3.07, p = 0.004); and experiencing an interruption in tuberculosis therapy (HR = 3.15, p = 0.002). The association between treatment interruption and risk of death was due to non-adherence during the intensive phase of treatment (HR = 3.20, p = 0.001). The median duration of treatment interruption did not differ significantly in either intensive or continuation phases between those who died and survived (23 versus 18 days, and 37 versus 29 days, respectively). No deaths were directly attributed to adverse drug reactions.

**Conclusions:**

In addition to advanced age, HIV and characteristics of advanced tuberculosis, experiencing an interruption in anti-tuberculosis therapy, primarily due to non-adherence, was also independently associated with increased risk of death. Improving adherence early during treatment for tuberculosis may both improve tuberculosis outcomes as well as decrease mortality.

## Background

Tuberculosis is a leading cause of death worldwide. According to the World Health Organization (WHO) over 1.7 million people with tuberculosis died in 2008 [1]. Advanced age, male gender, delays in diagnosis and treatment, drug resistance, and co-morbid conditions including HIV co-infection, diabetes, renal disease and COPD, have been associated with increased risk of death in patients with active tuberculosis [2-8]. A substantial proportion of deaths occur during tuberculosis treatment despite patients being on an appropriate regimen. Since drug-susceptible tuberculosis is a disease in which cure is achievable in a vast majority of patients (>95%) [9], it is plausible that the reported rate of mortality of 25.8 tuberculosis deaths/100,000 person years may be decreased if modifiable risk factors for mortality are identified and targeted early [10]. In this retrospective study, our principle objective was to evaluate outcomes of tuberculosis treatment in a low-incidence setting, where patients were managed at a tuberculosis control program without significant resource limitations. The period during which our study was conducted provides an opportunity to evaluate outcomes not only in HIV-uninfected patients but also a cohort of HIV-infected patients who were HAART treatment naïve, a population that is challenging to find in the United States but represents more than 90% of the estimated 1.4 million HIV-infected tuberculosis cases worldwide [1]. We previously reported our findings on risk factors for relapse of tuberculosis [11]. The current analysis examines the clinical, radiographic and microbiologic characteristics associated with increased risk of death during treatment for drug-susceptible pulmonary tuberculosis with the aim of alerting clinicians and tuberculosis control programs of characteristics that identify groups at increased risk for mortality.

## Methods

### Study Population

The current study of factors associated with mortality in drug-susceptible, pulmonary tuberculosis took advantage of a pre-existing dataset investigating risk factors for relapse [10]. For the parent study, we reviewed all tuberculosis cases reported to the San Francisco Tuberculosis Control Program from January 1, 1990, through December 31, 2001. Cases with initial drug resistance and those that were culture negative or non-cultured, with solely extra-pulmonary disease, treated outside the Department of Health, younger than 18 years of age, and cases diagnosed at autopsy were excluded. The focus of the parent study [11] was the influence of HIV co-infection on tuberculosis outcomes. Consequently, the parent study excluded foreign-born patients of self-reported Asian race due to their low rate of HIV infection compared with the general population of tuberculosis patients in San Francisco (1.5% versus 34.8%, respectively). For the current study, medical records were re-reviewed for additional details on therapy, morbidity and mortality. Of 700 cases in the parent study, medical records for 135 cases could not be recovered. Due to incomplete data, these cases were excluded from the final analysis. The final population for the current study was comprised of a cohort of 565 white, black, and US-born Asians, Pacific Islanders, Hispanic and non-Hispanic patients with culture-positive, drug-susceptible pulmonary tuberculosis.

### Study design

The study was approved by the Committee on Human Research of the University of California, San Francisco. A retrospective cohort study design was used to evaluate outcomes including mortality while on treatment for tuberculosis. A standard data collection form was used to record information extracted from all patient records, including HIV serostatus, history of opportunistic infections, tuberculosis, co-morbid conditions, chest radiograph results, bacteriology results, treatment regimen, use of directly observed therapy (DOT) and interruptions of treatment due to non-adherence or adverse reactions to tuberculosis medications. DOT was categorized as "full" (throughout treatment) or "partial" (during intensive phase of treatment only). San Francisco Tuberculosis Control Program requires treatment be initiated under DOT for patients with any of the following characteristics: sputum smear positive for acid fast bacilli, suspected or confirmed drug resistant disease, HIV co-infection, slow sputum conversion, homeless/shelter resident, history of intravenous or non-intravenous drug use, psychological disorder, alcohol abuse, history of prior tuberculosis, and when the treating clinician deems patient too infirm to self manage or suspects high likelihood of non-adherence. Viral load and CD4^+ ^T lymphocyte counts were provided by the San Francisco AIDS Registry. HIV status was unknown in 99 (17.5%) patients. As part of the parent study, rigorous evaluation of the medical records confirmed that these patients had no identifiable risk factors for HIV [11]. All patients received a rifamycin-based regimen for active pulmonary tuberculosis approved by the American Thoracic Society/Centers for Disease Control and Prevention/Infectious Diseases Society of America [9]. Rifabutin was used in place of rifampin in all patients receiving highly active antiretroviral treatment (HAART) and tuberculosis treatment.

### Definitions

Patients were considered cured if they completed all prescribed doses, converted their cultures to negative and had resolution of symptoms. Treatment failure was defined by positive cultures after 4 months of treatment [9]. Death was defined as any death that occurred during treatment for tuberculosis. Reported cause of death was determined based upon review of all available charts, death certificates and autopsy information. All deaths listed a cause in the medical records; in addition, 35 of the 37 deaths had death certificate and/or autopsy information. An episode of inadequate therapy was defined as an interruption in the prescribed regimen of any duration (prior to physician-approved treatment completion) that resulted in fewer than three anti-tuberculosis medications being administered during the initial intensive phase or fewer than two medications during the continuation phase for either of the following: 1) an adverse reaction to tuberculosis medications, or 2) an episode of patient non-adherence to one or more anti-tuberculosis medications.

All statistical analyses were performed using SAS Version 9.1 software (SAS Institute Inc., Cary, NC). Cox proportional-hazards regression analysis was used to assess risk factors for mortality. The chi squared test, Student's t-test, and Wilcoxon rank-sum test were used to test associations unrelated to mortality. Factors associated with mortality in univariate analysis (p < 0.10) were selected for a multivariate Cox proportional-hazards regression model. CD4 cell count was missing in 29 of 204 cases with HIV co-infection; for multivariate analyses involving CD4 count, missing values were imputed using a multiple imputation strategy [12]. All measured covariates were used in a regression on CD4 count run multiple times to estimate missing values. Kaplan-Meier survival analysis was performed to compare time to death by HIV status and treatment adequacy. Survival curves were compared using the Log-Rank test with follow-up time truncated at 18 months. A p-value of less than 0.05 was used as a threshold for significance.

## Results

Of 565 patients meeting eligibility criteria, 37 (6.6%) died while on treatment for tuberculosis. Sociodemographic and clinical characteristics are shown in Table 1. In univariate analyses, patients who died during treatment were more likely to have been older at time of diagnosis, to have been born in the US, to have been homeless within the year prior to tuberculosis diagnosis, to have required hospitalization at the time of their tuberculosis diagnosis, and to have a baseline positive sputum smear for acid fast bacilli (all p < 0.05). HIV-coinfection and having a history of an opportunistic infection other than tuberculosis were also more common in the group of patients who died (all p < 0.05). Median T-lymphocyte CD-4 count was lower at the time of diagnosis in HIV-coinfected patients who died during tuberculosis treatment as compared with HIV-coinfected patients who survived (68 versus 105 respectively, p = 0.06). Only one of the 24 HIV-infected patients who died received HAART during tuberculosis treatment; lack of HAART was highly associated with death (HR = 5.52), but due to limited numbers, the association did not reach statistical significance in univariate analysis (p = 0.10). Treatment under direct observation (DOT) was more commonly used in those who died (p = 0.04), however, this association was no longer significant in analyses controlling for HIV serostatus and sputum smear status, a reflection of the San Francisco Tuberculosis Control Program's strict policy to treat these high risk groups under DOT.

**Table 1:** Sociodemographic and clinical characteristics of patients by status at end of treatment by univariate Cox proportional-hazards regression (n = 565).

Characteristics		Death during treatment N (%)	Alive throughout treatment N (%)	p value
N		37	528	
Mean age at diagnosis ± SD		45.0 ± 16.2	40.7 ± 13.5	0.03
Median (Min-Max)		41 (22-88)	38 (18-94)	
Male Gender		29 (78.4)	423 (80.1)	0.95
Race	American Indian	0	9 (1.7)	0.99
	African American/Black	13 (35.1)	187 (35.4)	0.97
	Asian/Pacific Islander	1 (2.7)	10 (1.9)	0.73
	White	23 (62.2)	322 (61.0)	Ref
Hispanic ethnicity		7 (18.9)	159 (30.1)	0.16
US born		31 (83.8)	364 (68.9)	0.03
HIV positive		24 (64.9)	180 (34.1)	0.02
HAART during tuberculosis therapy		1/24 (4.2)	27/180 (15.0)	0.10
History of opportunistic infection		21/24 (87.5)	101/180 (56.1)	0.01
CD4 count				
≤ 200 cells/μL^1^		17/19 (89.5)	108/156 (69.2)	0.14
Median CD4 count at diagnosis (IQR)^1^		68 (34-104)	105 (47-269)	0.06
Received directly observed therapy^2^	Yes	28 (75.7)	251 (47.7)	0.02
	Partial*	4 (10.8)	90 (17.1)	0.67
	No	5 (13.5)	185 (35.2)	Ref
Episode of inadequate therapeutic regimen		25 (67.6)	161 (30.5)	0.01
During intensive phase^3^		24 (64.9)	129/525 (24.6)	0.001
Median duration of inadequate treatment (days) (IQR) (n = 153)		23 (6-35)	18 (9-34)	0.18
During continuation phase^4^		10/33 (30.3)	116/523 (22.2)	0.44
Median duration of inadequate treatment (days) (IQR) (n = 126)		37 (24-50)	29 (13-50)	0.24
Due to non-adherence		22 (59.5)	142 (26.9)	0.01
During intensive phase^3^		21 (56.8)	109/525 (20.8)	0.001
Median duration of inadequate treatment (days) (IQR) (n = 130)		8 (5-28)	15 (7-24)	0.13
During continuation phase^4^		10/33 (30.3)	108/523 (20.7)	0.64
Median duration of inadequate treatment (days) (IQR) (n = 118)		30 (16-50)	29 (14-53)	0.11
Due to adverse reaction^5^		10 (27.0)	49/527 (9.3)	0.10
During intensive phase^5^		7 (18.9)	39/527 (7.4)	0.21
Median duration of inadequate treatment (days) (IQR) (n = 46)		29 (8-39)	14 (6-38)	0.75
During continuation phase^5^		3 (8.1)	18/527 (3.4)	0.98
Median duration of inadequate treatment (days) (IQR) (n = 21)		25 (1-31)	14 (7-20)	0.40
Hospitalized for tuberculosis^6^		29 (78.4)	253/526 (48.1)	0.003
Homeless within 1 year of diagnosis^7^		16/27 (59.3)	138/356 (38.8)	0.04
Substance abuse at diagnosis		23 (62.2)	228 (43.2)	0.13
Sputum smear positive		27 (73.0)	233 (44.1)	0.004
Sputum culture positive		34 (91.9)	471 (89.2)	0.62
Extrapulmonary disease in addition to pulmonary disease		18 (48.7)	143 (27.1)	0.13
Cavitary disease		4 (10.8)	129 (24.4)	0.12
Any prior medical condition		25 (67.6)	288 (54.6)	0.13

1) Excluding 29 with missing data for CD4 cell count. 2) Excluding 2 with missing data for directly observed therapy. * Partial DOT = DOT used for less than the full duration of treatment (clinicians opted to transition select patients to self-administered therapy (SAT) at the end of the intensive phase of treatment, or initiated DOT in patients previously treated by SAT in whom clinical, radiographic or microbiologic response to treatment was delayed). 3) Excluding 3 with missing data for intensive phase. 4) Excluding 9 with missing data for continuation phase. 5) Excluding 1 with missing data for adverse reaction. 6) Excluding 2 with missing data for hospitalization. 7) Excluding 182 with missing data for housing status.

An episode of interruption in therapy from any cause was identified in 205 of 565 study participants. Of the 205, 186 cases had an interruption in therapy that met the study definition for inadequate therapy. Overall, 27% of patients experienced an episode of inadequate therapy during the intensive phase of treatment, and 23% in the continuation phase. Experiencing an episode of inadequate therapy, in particular during the intensive phase, was significantly more common in the patients who died as compared with those who survived (p = 0.001). Analyzed separately by the two causes defined for inadequate therapy, non-adherence versus adverse reactions, the association remained significant for non-adherence during the intensive phase (HR = 3.20, 95% CI 1.64-6.24, p = 0.001), but not for adverse reactions. While the relative hazard was high for the association between adverse reactions during the intensive phase and mortality, the relationship did not reach statistical significance (HR = 1.74, 95% CI 0.73-4.13, p = 0.21). Overall, the median duration of treatment interruption for patients who died was not different from those who survived (37.5 days versus 36 days, respectively, data not shown). Examining the median duration of days a patient received inadequate treatment in more detail, by phases of treatment (Table 1), we found that both intensive and continuation phase interruptions were longer in those who died (median of 23 and 37 days, respectively) as compared with those who survived (median of 18 and 29 days, respectively). Whereas the association between median duration and mortality was in a biologically plausible direction when looked at by phases of treatment, the differences were not statistically significant (all p > 0.1).

Of the 37 deaths during treatment for tuberculosis, 12 (32.4%) had tuberculosis listed in the death certificate, autopsy or chart either as the direct cause of or contributing to cause of death, with nine of these also having AIDS listed as a contributing factor. Of the remaining 25 deaths for which tuberculosis was not listed in the death certificate or autopsy, 13 (35.1%) were attributed to complications of AIDS. None of the deaths were directly attributed to adverse drug reactions or hepatotoxicity. In multivariate analysis, four characteristics were independently predictive of mortality: HIV co-infection (HR = 2.57, 95% CI 1.17-5.64, p = 0.02); older age at diagnosis (HR = 1.52 per 10 years, 95% CI 1.18-1.95, p = 0.001); initial sputum smear positive for acid fast bacilli (HR = 3.07, 95% CI 1.44-6.56, p = 0.004); and episode of inadequate tuberculosis therapy during the intensive phase of treatment (HR = 3.15, 95% CI 1.52-6.52, p = 0.002) (Table 2). Being born in the US was associated with an increased risk for death, but did not reach our predetermined level of statistical significance (HR = 2.70, 95% CI 0.96-7.63, p = 0.06). A multivariate analysis of HIV-infected patients was performed separately, and five characteristics were independently predictive of mortality: history of opportunistic infection (HR = 6.24, 95% CI 1.26-31.0, p = 0.03); lack of HAART while on tuberculosis therapy (HR = 9.07, 95% CI 1.06-77.4, p = 0.04); US birth (HR = 7.79, 95% CI 1.10-55.1, p = 0.04); positive sputum smear (HR = 4.38, 95% CI 1.43-13.4, p = 0.01); and episode of inadequate therapy during the intensive phase (HR = 3.47, 95% CI 1.27-9.50, p = 0.02) (data not shown). CD4 cell count at the start of therapy was included in the multivariate model, however, its protective association was not statistically significant (HR = 0.95 per 25 cells, 95% CI 0.84-1.06, p = 0.47).

**Table 2:** Predictors of death during treatment (n = 565).

Predictors		Death during treatment N (%)	Unadjusted HR (95% CI), p value	Adjusted HR (95% CI), p value
N		37		
Mean age at diagnosis ± SD (hazard/10 year increase)		45 ± 16	1.26 (1.03-1.55), 0.03	1.52 (1.18-1.95), 0.001
Gender	*Male*	29 (6.4)	0.98 (0.44-2.14), 0.95	
	*Female*	8 (7.1)		
Ethnicity	*Hispanic*	7 (4.2)	0.55 (0.24-1.27), 0.16	
	*Non-Hispanic*	30 (7.5)		
Place of birth	*U.S*.	31 (7.9)	2.92 (1.10-7.74), 0.03	2.70 (0.96-7.63), 0.06
	*Foreign*	6 (3.5)		
HIV status	*Positive*	24 (11.8)	2.30 (1.14-4.61), 0.02	2.57 (1.17-5.64), 0.02
	*Negative/Unknown*	13 (3.6)		
DOT^1^	Yes/Partial*	32/373 (8.6)	2.65 (1.02-6.87), 0.04	1.31 (0.47-3.64), 0.61
	*No*	5/190 (2.6)		
Episode of inadequate therapy during intensive phase^2^	*Yes*	24/153 (15.7)	3.39 (1.68-6.83), 0.001	3.15 (1.52-6.52), 0.002
	*No*	13/409 (3.2)		
Episode of inadequate therapy during continuation phase^3^	*Yes*	10/126 (7.9)	0.72 (0.32-1.65), 0.44	
	*No*	23/430 (5.4)		
Sputum smear status	*Positive*	27 (10.4)	2.97 (1.43-6.17), 0.004	3.07 (1.44-6.56), 0.004
	*Negative*	10 (3.3)		
Extrapulmonary in addition to pulmonary	*Yes*	18 (11.2)	1.69 (0.85-3.34), 0.13	
	*No*	19 (4.7)		
Cavitary disease	*Yes*	4 (3.0)	0.43 (0.15-1.23), 0.12	
	*No*	33 (7.6)		
Any prior medical condition	*Yes*	25 (8.0)	1.74 (0.85-3.54), 0.13	
	*No*	12 (4.8)		

1) Excluding 2 with missing data for directly observed therapy. * Partial DOT = DOT used for less than the full duration of treatment (clinicians opted to transition select patients to self-administered therapy (SAT) at the end of the intensive phase of treatment, or initiated DOT in patients previously treated by SAT in whom clinical, radiographic or microbiologic response to treatment was delayed). 2) Excluding 3 with missing data for intensive phase. 3) Excluding 9 with missing data for continuation phase. Note-HR = hazard ratio

**Figure 1:**
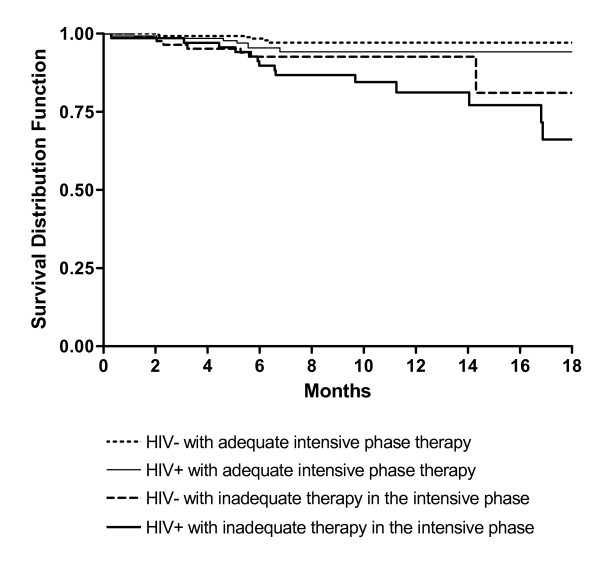
**Kaplan-Meier survival curves showing time to death by HIV status & treatment adequacy during the intensive phase of therapy**.

Survival curves generated using the Kaplan-Meier method stratified by HIV status and adequacy of intensive phase therapy with follow-up time truncated at 18 months (overall p = 0.001) are shown in Figure 1. The greatest difference in rates of survival were between patients with both HIV co-infection and an episode of inadequate therapy (89.7% survival at 6 months, 81.2% at 12 months), compared with patients with neither HIV co-infection nor inadequate therapy (98.4% survival at 6 months, 97.1% at 12 months) (p < 0.001). Survival rates between those with HIV co-infection and no evidence of inadequate therapy (95.4% survival at 6 months, 94.2% at 12 months) and those without HIV infection who had an episode of inadequate therapy (92.6% survival at 6 and 12 months) were the same (p = 0.41).

## Discussion

In this study, four characteristics were independently associated with increased risk of mortality during treatment for drug susceptible tuberculosis. These characteristics were older age at diagnosis, HIV co-infection, sputum smear positivity for acid fast bacilli at baseline and experiencing an interruption in tuberculosis therapy during the intensive phase of treatment. Whereas prior studies have reported older age, comorbid conditions including HIV and advanced tuberculosis as non-modifiable determinants of death in tuberculosis [4,13-15], our study identifies treatment interruptions as a risk factor that is potentially modifiable through programmatic action. The association is also important to recognize as it suggests that interruptions in therapy have implications that go beyond recognized increases in risk for microbiologic failure, relapse of disease or even development of drug resistance.

We sought to clarify other aspects related to interruptions, including the timing, duration and cause of interruptions. The timing of inadequate therapy for tuberculosis proved to be important. Interruptions during the intensive phase were associated with mortality, whereas interruptions during the continuation phase were not. This finding suggests that interruptions occurring early, when drugs have bactericidal activity against replicating organisms, have greater implications for risk of death than interruptions during the continuation phase when therapy is focused on clearing persistent bacilli to prevent relapse [16]. In regard to duration of interruptions, the median duration was longer in those that died as compared with those that survived, however, the difference was not statistically significant in either intensive or continuation phases. Next, we explored the two causes of interruptions in treatment, adverse reactions and non-adherence, and found that non-adherence appeared to be driving the association. These stratified analyses may have lacked power to definitively exclude adverse reactions as a risk factor for death, however, we believe that differences between the two causes of interruption make this distinction plausible. In reviewing the treatment interruptions, we found that interruptions from non-adherence often resulted in complete cessation of all medications, in contrast to interruptions from adverse reactions which were handled by temporary cessation of medications followed by swift rechallenge until a tolerable regimen was identified. We also reviewed autopsy reports, death certificates and the medical charts for all 37 patients who died during treatment and found no evidence to suggest that adverse drug reactions played a direct role in any of the deaths. Finally, it is important to note that whereas we found non-adherence to be associated with increased risk for death, the reverse relationship seen between DOT use and death was due to confounding by association given that our program has a strict policy to initiate DOT in all patients at high risk for poor outcomes, including death.

Our study has several limitations. Given its retrospective nature, we were unable to distinguish between the reason for and effect of treatment interruption as it related to the association with death. While we reviewed all available charts, computer records, death certificates, and autopsy information to determine cause of death, a minority of cases had post mortem analyses (7/37). Consequently, factors associated with death were not necessarily causal. Second, our findings are not relevant for HAART treated/exposed populations, as the majority of the HIV-infected individuals in this study were HAART naive. Of the 37 deaths, only one HIV-infected individual was on HAART, precluding our ability to evaluate the role of HAART in preventing death. This is an important limitation given the well recognized benefits of HAART on survival. However, we believe our findings are still relevant as the majority of HIV-related tuberculosis in the world continues to be managed without early initiation of HAART. Finally, we cannot extrapolate these findings to foreign-born individuals who self-reported Asian race as they were excluded from the parent study. A recent evaluation of deaths among 7,999 culture positive pulmonary tuberculosis cases in Shanghai, China, identified advanced age, sputum smear positivity, comorbid conditions and gender as factors associated with death [15]. However, additional studies are needed to determine whether these factors also pertain to foreign-born Asians residing in the US.

## Conclusion

In summary, in addition to the nonmodifiable characteristics of advanced age at diagnosis, HIV co-infection and sputum smear positivity at baseline, experiencing an interruption in treatment during the intensive phase of treatment was associated with an increased risk of death. Our analyses suggest that non-adherence, more so than adverse drug reactions, may be driving this association. Continued investment in programs such as DOT that can promote adherence early during treatment for tuberculosis may both improve tuberculosis outcomes as well as decrease mortality.

## Competing interests

The authors declare that they have no competing interests.

## Authors' contributions

PN participated in design of the study, conducted chart reviews for data abstraction, and drafted the manuscript. IR, BCDJ, AU participated in chart review, data abstraction and interpretation of data. LGH and DHO participated in the design of the study and performed the statistical analyses. LMK and PCH participated in data interpretation and manuscript revisions. CLD conceived of the study, and participated in its design and coordination. All authors read and approved the final manuscript.

## Pre-publication history

The pre-publication history for this paper can be accessed here:

http://www.biomedcentral.com/1471-2334/11/1/prepub
